# UPObase: an online database of unspecific peroxygenases

**DOI:** 10.1093/database/baz122

**Published:** 2019-12-09

**Authors:** Muniba Faiza, Dongming Lan, Shengfeng Huang, Yonghua Wang

**Affiliations:** 1 School of Food Science and Engineering, South China University of Technology, Wushan road, Tianhe district, Guangzhou 510640, Guangdong province, China; 2 Laboratory for Marine Biology and Biotechnology, Pilot National Laboratory for Marine Science and Technology (Qingdao) 1 Wenhai road, Aoshanwei, Jimo, Qingdao, Shandong, 266237, China; 3 State Key Laboratory of Biocontrol, School of Life Sciences, Sun Yat-Sen University, No., 135, Xingang Xi road, Guangzhou, 510275, China

## Abstract

There are many unspecific peroxygenases (UPOs) or UPO-like extracellular enzymes secreted by fungal species. These enzymes are considered special in their ways of catalyzing a wide variety of reactions such as epoxidation, peroxygenation and electron oxidations. This enzyme family exhibits diverse functions with thousands of UPOs and UPO-like sequences. These sequences are difficult to analyze without proper management tool and therefore desperately calls for a unified platform that can aide with annotation, classification, navigation and easy sequence retrieval. This prompted us to create an online database called Unspecific Peroxygenase Database (UPObase) (upobase.bioinformaticsreview.com) which currently includes 1948 peroxygenase-encoding protein sequences mined from more than 800 available fungal genomes. It provides information such as classification and motifs about each sequence and has functions such as homology search against UPObase sequence analyses such as multiple sequence alignments and phylogenetic trees. It also provides a new sequence submission facility. The database has been made user-friendly facilitating systematic search and filters. UPObase allows users to search for the sequences by organism name, cluster ID and accession number. Notably, in our previous study, 113 UPOs were classified into five subfamilies (I, II, III, IV and V) and an undetermined group (Pog) which remain established. In this study, using 1948 UPOs in our database, we were able to further identify six novel sub-superfamilies (Pog-a, Pog-b, Pog-c, Pog-d, Pog-e and Pog-f) with signature motifs and two distinct groups in Subfamily I and III, Ia and Ib, IIIa and IIIb, respectively. With the novel UPO-like sequences and classification, UPObase may serve for researchers working in the area of enzyme engineering and related fields.

## Introduction

Unspecific peroxygenases (UPOs) (EC 1.11.2.1) represent the oxidoreductase sub-subclass of heme-thiolate proteins obtained from fungal species (1). Fungal UPOs catalyze a wide variety of reactions such as epoxidation, dealkylation, hydroxylation, one- and two-electron oxidations and oxidation of aromatic and heterocyclic compounds, inorganic halides and organic heteroatoms (2–4). Fungal UPOs are considered as intriguing enzymes because of their various significant properties such as stability, specificity, catalytic activity, high specific activity, water-soluble nature and capability of catalyzing reactions using inexpensive peroxides and cofactors such as Mg^2+^. Therefore, the UPOs are also termed as the ‘ideal biocatalysts for (sub)-terminal hydroxylation of short-chain and medium-chain alkanes under mild conditions’ (5).

Some UPOs which are known to date with experimental evidence include *Agrocybe aegerita* UPO (*Aae*UPO), *Marasmius rotula* (*Mro*UPO) and *Coprinellus radians* (*Cra*UPO), among which the protein crystal structure of *Aae*UPO (2YOR) and *Mro*UPO (5FUJ) is only available to date. UPOs are classified as heme-thiolate peroxidases (HTPs) due to their heme-ligation bond with *cysteine* and their similarity with other HTPs known as chloroperoxidases (CPOs). CPOs exhibit strong peroxidase activity but show less peroxygenase activity. There are existing known conserved motif patterns responsible for the catalytic activities of UPOs and CPOs (i.e. -PCP-EGD-R-E and -PCP-EHD-E, respectively) (6,7). However, in the preliminary publication, UPOs have been classified on the basis of phylogeny and sequence motifs, into five subfamilies and a superfamily which includes *Mro*UPO and some CPOs showing an intermediate behavior between the peroxygenases and peroxidases (8). But there are many other UPOs or UPO-like sequences which were not included in the previous analysis and thereby not classified under any known subfamilies and superfamily.

UPOs are considered intriguing enzymes, which could also possess some other necessary functions which may have not been discovered yet due to their limited information. There are many UPOs existing in the fungal kingdom with a wide range of activities but lack a proper classification and annotation for their systematic analysis. Therefore, in this study, core sequences of UPOs obtained in the previous study are used to search for more UPOs and organized into a proper classified system. Further, on the basis of new data obtained, the sequences are subclassified based on their phylogeny and sequence motifs, thereby constituting the Unspecific Peroxygenase Database (UPObase).

Sequence databases such as GenBank (9), Ensembl Fungi (10), MycoBank (11), EPPO-Q-Bank (12) archive information on nucleotide and protein sequences. Specialized databases use them as primary data, for instance, Pfam (13) which classifies sequences into families. Similarly, UPObase is a more specialized database consisting of protein sequences obtained by genome mining of all fungal genome sequences present in Ensembl Fungi (10). The sequences have been classified into new subfamilies and superfamilies based on their phylogenetic studies and motif patterns in their sequences. Some other enzyme-based databases exist such as Lipase Engineering Database (14) which provides information about lipases including their sequences and structures, PeroxiBase (15) which is a peroxidase database which is dedicated to peroxidases and other oxidoreductase enzymes and MEROPS (16) which is dedicated to peptidases. However, the comprehensive enzyme information system called BRENDA (17) is composed of multiple enzymes including their nomenclature and inhibitors but lacks information on UPOs. Any single database dedicated to the UPO enzyme is not available to date which can provide sequence details, submission portal and real-time sequence analyses. UPObase is the only all-UPO protein sequence database designed to perform a systematic analysis of sequence, function and phylogenetic relationships for these extracellular proteins found in fungi. Besides, this database provides more sequences along with detailed information which may help in discovering new potential functions of UPOs and study their physiological role in fungi. The sequences in UPObase are assigned to their corresponding subfamilies and superfamilies along with their signature motif patterns for their easy identification.

## Methods

### Genome sequence retrieval

A set of fungal genomes constituting 812 different species (or strains) were downloaded from the Ensembl Fungi genome database via FTP (ftp://ftp.ensemblgenomes.org/pub/) (10). The genome sequences consist of a large number of peptide sequences. These sequences were used as primary data which were further subjected to mining composed of various filters.

### Phylogenetic analysis

The phylogenetic analysis was carried out using MEGA7 software (18). A best-fit model to the data was selected using the PROTTEST3 (20) program. It recommended WAG+G+F, namely, WAG (19) amino acid substitution matrix, gamma distribution (under four rate categories) and empirical amino acid frequencies. Maximum likelihood trees were constructed with a bootstrap replicate of 300 using the same model.

### Real-time sequence analyses

The multiple sequence alignments (MSAs), phylogenetic trees and their corresponding percent identity matrix (PIM) are generated in real time for each user query. A simple MSA was generated using Clustal Omega (21), and color-coded alignment was generated using MUSCLE (22). It uses Erik Sonnhammer’s Belvu Editor (23) to color the alignment. The phylogenetic neighbor-joining (NJ) trees and their corresponding PIM are generated using ClustalW2 (24), and PhyD3 JavaScript (25) has been implemented for tree visualization in the form of phylogram or dendrogram.

### Database construction

**Figure 1:**
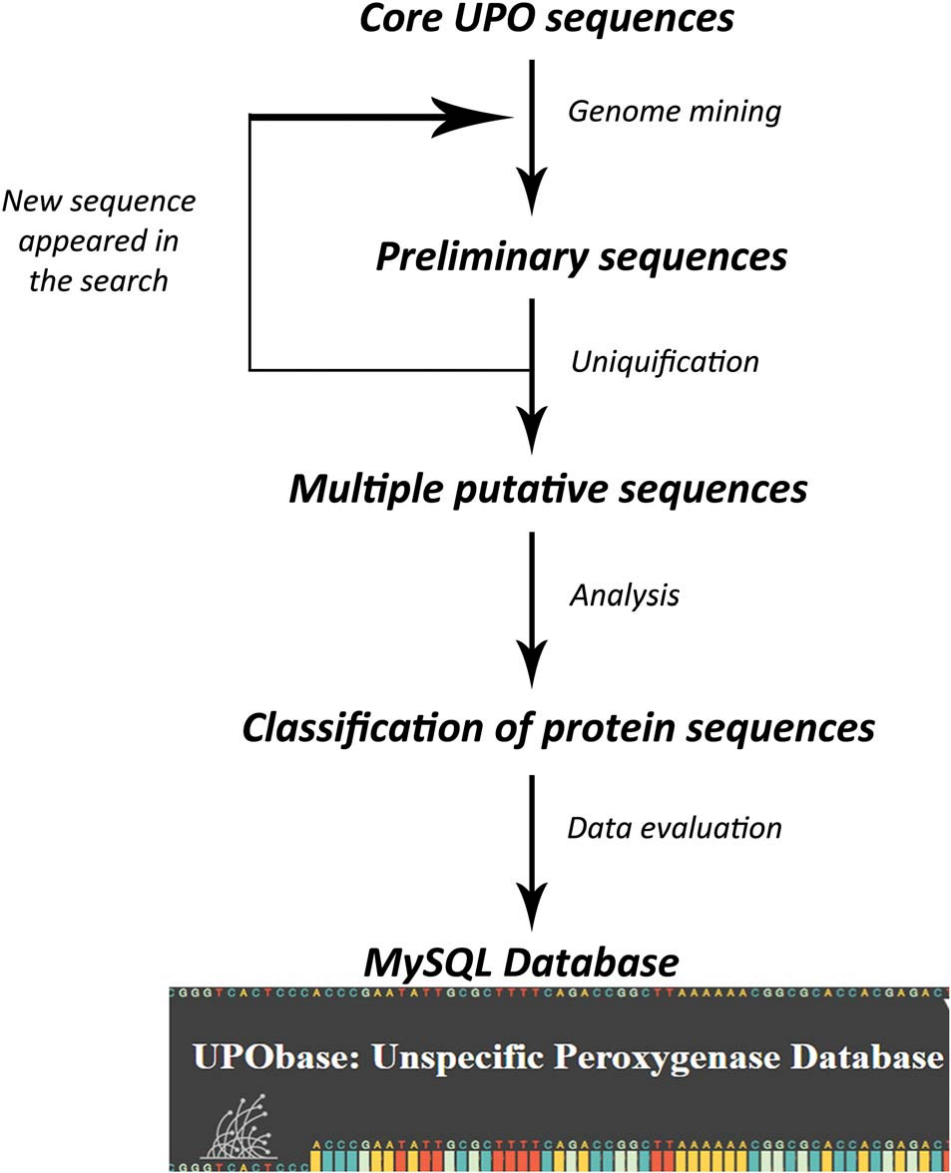
A scheme involved in the database development process.

A set of previously found 113 UPO encoding sequences belonging to different subfamilies and a superfamily were used to find more sequences using an improved pipeline which we created on the basis of our previous study. The database construction is based on an iterative process of searching for UPO encoding sequences for each and every new sequence that appeared in preliminary searches (Figure 1). In the first round, each of the core UPO sequences was used as a query for similarity search using PHMMER (http://hmmer.org; version 3.1b2) against the generated fungal genome database with an E-value and an inclusive E-value set to 10.0 and 0.01 respectively, providing ~1 false positive in every 100 searches. The output sequences were clustered using cd-hit software (26) at the 90% similarity cutoff and a word length of five residues. The resultant sequences were further clustered using graph-based clustering software MCL (27) at an inflation value set to 1.4. The obtained clustered sequences were then searched for sequence motifs corresponding to their subfamily type resulting in a large number of sequences. This step is repeated for each new sequence that appeared in the similarity search. In the second round, in order to reduce the redundant sequences, the resultant sequences were subjected to sequence-based clustering again at a 95% similarity cutoff providing a total of 1948 clusters. The representative sequences from each cluster which represent the operational taxonomical units (OTUs) were selected and then further analyzed which resulted in the reclassification of UPOs. Finally, we obtained 1948 total UPO encoding sequences (including *Aae*UPO, *Mro*UPO and *Lfu*CPO) constituting the database.

## Results

### Sequence identification

To create the UPObase, we used a pipeline to search for UPOs and UPO-like sequences. This pipeline involves homology search refined with various filters such as blast, sequence-based and graph-based clustering and motif search. Additionally, the filtered sequences were again subjected to sequence-based clustering coupled with phylogenetic analysis in order to remove non-UPO sequences. Therefore, these sequences represent a complete and reliable set of UPOs or UPO-like protein sequences obtained from an *in silico* filtering including clustering, motif search and phylogenetic analysis. After a thorough sequence and phylogenetic analyses, these sequences were found to be exhibiting different motif patterns which led to their subclassification. The main purpose of UPObase is to provide a unified platform for systematic analysis of UPOs. Currently, the database consists of a complete set of 1948 protein sequences of UPOs or UPO-like extracted from 812 fungal genomes.

### Database architecture

**Figure 2:**
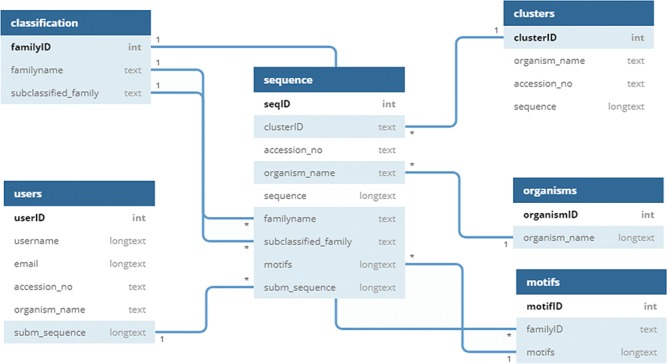
Schema of UPObase.

UPObase is a relational MySQL database, and its complete architecture is explained in Figure 2. It involves two different layers of sequences: UPOs and UPO-like sequences (thousands of sequences) > clustered highly similar sequences (1948 sequences with 95% sequence similarity). This helped to remove the redundant and insignificant sequences from the database. These two layers are linked together with the cluster IDs. Each cluster consists of various sequences sharing 95% and above similarity (layer 1), and a representative sequence from each cluster is selected for the next layer of sequences (layer 2).

The information regarding classification, motifs, organisms, and sequences is stored in separate tables linked to each other. The clusters with a specific ID are stored in a table, and only the representative sequence (OTU) is added in the sequence table with a linked cluster ID. The motifs are linked with the family and sequence tables where a motif pattern is assigned for each sequence depending upon its classification. The user-submitted sequences and other related information is stored in a separate table which will be added into the sequences after the validation and classification.

### Web interface

**Figure 3:**
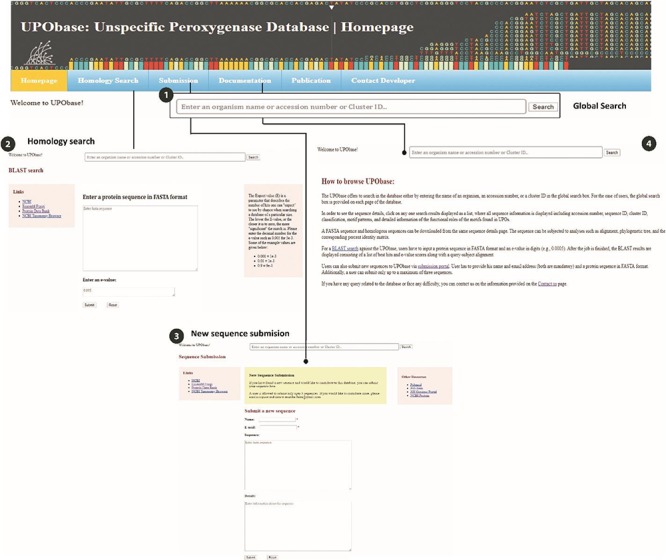
An overview of the utilities of UPObase. (1) A global search box displayed at every page of the database to allow browsing convenient; (2) BLAST search feature where a user can enter any sequence and find homologous sequences corresponding to the input; (3) a new sequence submission portal; and (4) documentation page for help.

**Figure 4:**
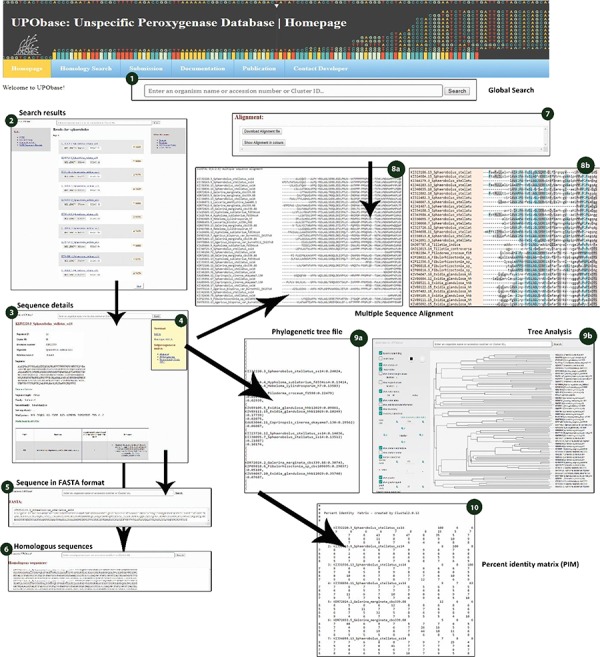
Sequence details displayed for each and every sequence searched within UPObase. (1) the global search box; (2) search results displayed as a list to each search term; (3) sequence details; (4) download and subjecting sequence to analyses options; (5) sequence displayed in FASTA format; (6) FASTA sequences of the homologs corresponding to the sequence; (7) download files for alignment, tree and PIM; (8), (9) and (10) real-time created MSA, phylogenetic tree and PIM, respectively.

The UPObase is available online at upobase.bioinformaticsreview.com, and its complete web interface is explained in Figure 3. The webpages can be easily accessed on any PHP and JavaScript supporting web browsers. A global search bar is given on each page to allow users to browse the database by any organism name, accession number or cluster ID which provides a list of entries in the database along with its sequence length and a direct link to download its FASTA sequence (Figure 4). A user can easily get all the information about any sequence by clicking the link. The details for each sequence include sequence ID, cluster ID, accession number, organism name, database source (from where the genome was downloaded), the sequence and the sequence features including sequence length, family, sub-subfamily, motif pattern and the tables which describe the functional role of motifs in detail. The sequence FASTA and corresponding homologous FASTA can be downloaded from the section provided in the right (Figure 4 (2)). In order to study the relationship among the other UPO-encoding sequences, real-time generated alignments and phylogenetic trees of each sequence are provided. The similarity among the homologous sequences can be seen in the real-time generated PIM corresponding to the alignment and the tree. Documentation provides information on browsing the database. In case of any difficulty, users can contact by sending an email provided at the contact information page.

**Figure 5:**
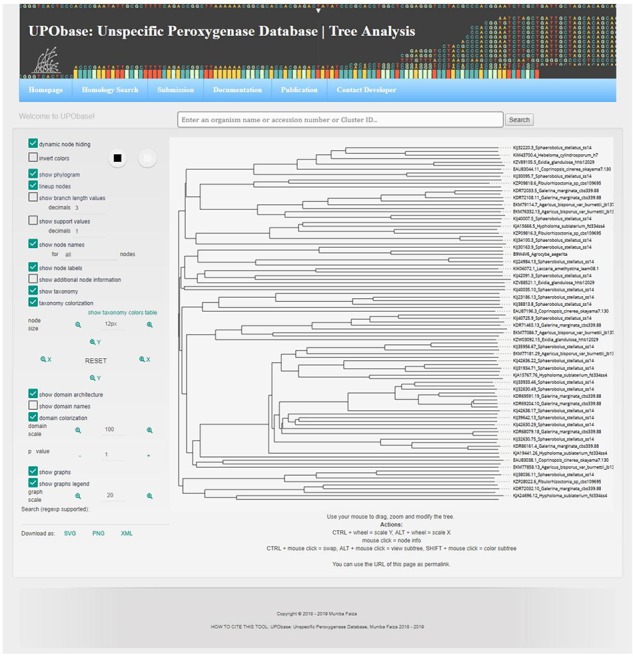
Tree analysis showing various key features.

### Database utility

#### Sequence retrieval

The sequences from UPObase can be easily retrieved either by entering an organism name, or accession number or a cluster ID. As shown in Figure 4, if a user searches for a term, for example, ‘*Sphaerobolus*’, as a result, it will provide a complete list of the given entries in the database along with their sequence ID, sequence length and a direct link to download its FASTA sequence (Figure 4 (2)). The FASTA sequence and corresponding homologous FASTA sequences can also be downloaded from the sequence details page via the links given in the top right corner (Figure 4 (4)). If a user searches UPObase by providing a cluster ID, sequences belonging to that cluster will be displayed as a list, and it may include different organisms. If a user browses by an accession number, only a sequence linked to this accession number will be displayed. In case of any difficulty, users can refer to the examples for browsing UPObase that are provided on the documentation page with screenshots. The corresponding homologous sequences in FASTA format can be downloaded by exploring these entries displayed as a result of the search.

#### Sequence information

Each sequence in UPObase is stored with its complete information including its classification, motif pattern, sequence ID and cluster ID. All the information is displayed for each entry in the database along with the tables illustrating the functional roles of motif patterns found in all UPOs (Figure 4 (3)). This helps to identify the functional roles (either proved or hypothesized) of sequences belonging to different sub-subfamilies and sub-superfamilies. The conserved sequence patterns may also help in designing family-specific primers for screening new enzymes. The properly classified sequence information makes easy to further study their functional roles and to describe reasons behind their intriguing behavior.

#### Homology search

The database sequences can be searched and compared with any other enzyme using the homology search which may help in the prediction of possible functions of unknown proteins. Users can adjust the e-value for the blast search against the database according to their requirements as shown in Figure 3 (2). The most relevant BLAST hits are displayed as output which consists of the subject query alignment along with e-value, bit score, percent identity and length of the subject sequence.

#### Sequence submission

New sequences can be submitted via the submission portal where a user has to provide his name, email address and details about the new sequence including the source and type of the sequence whether hypothesized or expressed (Figure 3 (3)). A single user can submit a maximum of three sequences to the database. If users wish to submit more sequences, then they can send a request and data to the email address mentioned at the contact information page on the website. The criterion to submit any sequence in UPObase includes the following: sequence can be hypothetical or expressed, must belong to the fungal kingdom and must be longer than 100 amino acid residues. The user-submitted sequences will be added into the database after manual curation and validation. The curation involves motif pattern search to identify the subfamily/superfamily and classification of the organism. This new sequence submission portal allows the UPObase to grow and helps in making available all the new sequences discovered so far.

**Figure 6:**
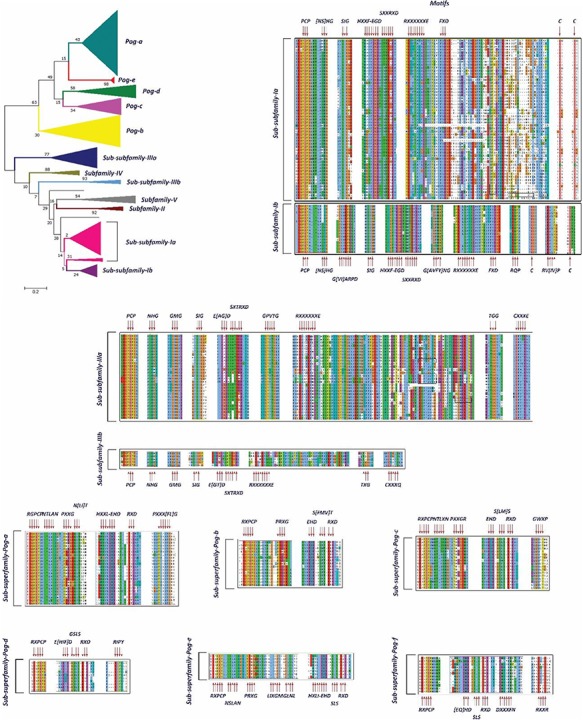
A phylogenetic tree and MSAs of UPO encoding sequences belonging to sub-subfamilies and sub-superfamilies which are reclassified. The motifs specific to each sub-subfamily are signified with a red arrow.

#### Sequence analyses

A sequence in the database can be easily subjected to analysis by creating MSA with the other corresponding homologous sequences present in UPObase. Phylogenetic analyses can also be carried out, and in order to identify the similarity amongst these sequences, a PIM is also generated. In order to include the new and updated sequences in the analysis, the generation of MSA, phylogenetic tree and PIM is completely automated. In addition, the MSA can be visualized in a color scheme showing the conserved residues (Figure 4 (8b)). The generated phylogenetic trees can be analyzed in the form of a phylogram or a dendrogram with various other visualization options (Figure 4 (9b)). Phyd3 offers various features to analyze Newick and XML tree files including information for each node in the tree, visualize branch lengths, support values, adjust the graph, see the graph in different background and foreground colors and display/hide node names and labels (25). The tree graph can be exported in SVG, PNG and XML format (Figure 5).

In summary, UPObase has been designed to study and analyze all fungal UPOs but it also works as a platform to perform similarity search and comparison of any other enzyme of interest with the UPOs. The conserved patterns and classification of UPObase can also be used for identifying functions for the unknown proteins. Besides, these discovered new members in the families may reveal some novel characteristics in addition to those exhibited by the UPOs.

**Figure 7:**
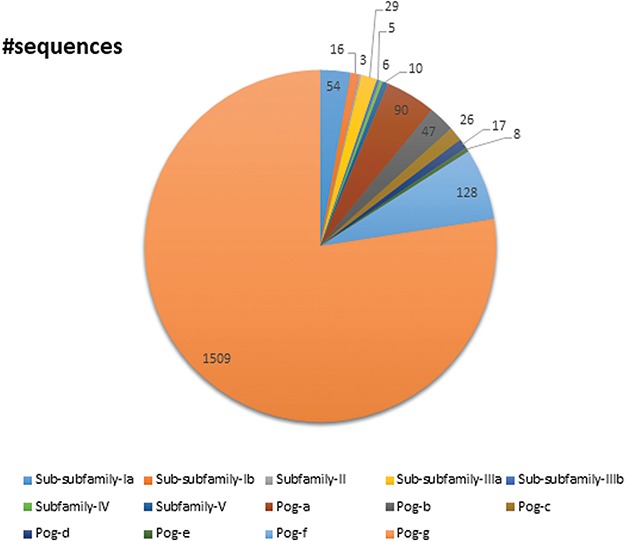
A pie chart showing the total number of sequences present in the database classified into subfamilies and superfamilies.

### Classification of UPOs

In our preliminary work, we found 113 putative UPO sequences, which were classified into five different subfamilies (I, II, III, IV and V) and a superfamily (Pog) based on the motifs present in their sequences and the phylogenetic analysis. Here, in this study, we have found 20 times more UPO and UPO-like sequences at our disposal. Previously, Subfamily I was found to have a specific motif pattern (Table 1). Based on the current data of UPOs, a new slightly different motif pattern has been found to exist in this subfamily, and hence, it is subclassified into two sub-subfamilies: Sub-subfamily Ia having the former motif pattern and Sub-subfamily Ib with a newly found motif pattern (Figure 6). However, some motifs such as [NS] HG, SIG and SXXTRXD which were present in all UPOs are still present in the new sub-subfamilies. After re-clustering in the second step, Subfamily II remains with a very few numbers of OTUs and not further subclassified, which is found to have the same motif pattern as explained previously. According to the phylogenetic and sequence analyses of Subfamily III sequences, it has been classified into two new sub-subfamilies: Sub-subfamily IIIa and Sub-subfamily IIIb (Figure 5). These two sub-subfamilies were found to have some additional motifs in their sequences to the pattern explained previously (Tables 1 and 2). The Subfamily IV and Subfamily V UPO encoding sequences consisted of the same motif pattern as explained previously (Table 1). No new motif pattern was found to exist in these sequences. However, the Pog superfamily which was previously not found to be consisting of any signature motif, after finding more sequences belonging to this superfamily, led to its subclassification into seven sub-superfamilies based on the phylogenetic tree and the sequence motifs (Figure 5). The hypothesized functions of the newly found motifs are explained in Table 2 to allow users to identify the possible roles of subfamilies and superfamilies.

**Table 1:** represents the motif patterns specific to sub-subfamilies and sub-superfamilies.

**Subfamily/superfamily**	**Sub-subfamily/sub-superfamily**	**Motif pattern**
Subfamily-I	Sub-subfamily-Ia	PCP—[NS]HG—SIG—HXXF—EGD—SXXRXD—RXXXXXXE—FXD—C—C
	Sub-subfamily-Ib	PCP—[NS]HG—GVARPD—SIG—HXXF—EGD—SXXRXD—G[AVFY]NG—RXXXXXXE—FXD—RQP—C—RV[IV]P—C
Subfamily-II	-	PCP—NHG—RGN—S[IL]G—VPPLPG—IDG—HGRF—EGD—SMTRXD—RXXXXXXE—TXXXXXXR
Subfamily-III	Sub-subfamily-IIIa	PCP—NH[NG]—G[ML]G—SIG—E[GA]D—SXTRXD—GPXTG—RXXXXXXE—TGG—CXXXE
	Sub-subfamily-IIIb	PCP—NH[NG]—G[ML]G—SIG—E[GT]D—SXTRXD—RXXXXXXE—TXG—CXXXQ
Subfamily-IV	-	PCP—N[HY][NG]—FXXXD—S[IL]G—CDA—HXXF—EGD—SLTRXD—RXXXXXXE—GAAXXXYE
Subfamily-V	-	EDXXH—PCP—NHG—SIG—GXG—EGD—SVTRXD—RXXXXXXE
Pog-superfamily	Pog-a	RGPCP—NTL[AT]N—PXXG—NXT—HXXL—EHD—RXD—PXXXFG
	Pog-b	RXPCP—PRXG—[EQ]HD—S[FMV]T—RXD
	Pog-c	RXPCP—NTLXN—PXXGR—EHD—S[ML]S—RXD—GWXP
	Pog-d	RXPCP—E[IHF]D—GSLS—RXD—RIPY
	Pog-e	RXPCP—NSLAN—PRXG—LIXGM—GLNL—HXLI—EHD—SLS—RXD
	Pog-f	RXPCP—[EQ]HD—S[LM]S—RXD—DXXXFN—RXXR
	Pog-g	No signature motif

**Table 2:** summarizes the hypothesized functions of the preliminary and newly found subfamilies and/ sub-subfamilies and sub-superfamilies.

**Subfamily**	**Sub-subfamily/ superfamily**	**Motif**	*Roles of amino acids present in the motif	**Hypothesized functions of the subfamily/superfamily**
**I**	Ia	FXD	*Phe* is basically involved in stacking interactions with other aromatic side-chains and the *Asp* is frequently involved in salt-bridges interacting with positively charged amino acids to create stabilizing H-bonds which can be important for proteins stability.	may actively involve in interacting with aromatic residues and in forming stable H-bonds imparting to the structural stability, and in substrate recognition.
		*Cys-Cys*	the disulfide bond is mostly involved in providing stability to protein structure.
	Ib	GVARPD	*Gly* provides the conformational stability; *Val* may play a role in substrate recognition; *Ala* is involved in substrate recognition and specificity; *Arg* is frequently involved in making salt-bridges with the negatively charged amino acids creating stable H-bonds which may be crucial for the structure stability; *Pro* plays an important role in molecular recognition; and Asp residues create a stable H-bonds.
		G[AVFY]NG	Again *Gly* provides the conformational stability; *Tyr* and *Phe* make stacking interactions with the aromatic side chains; the *Asn* is involved as proteins active and binding sites.
**II**	-	RGN	*Arg* is frequently involved in making salt-bridges with the negatively charged amino acids creating stable H-bonds which may be crucial for the structure stability; the *Gly* provides the conformational stability, and the *Asn* is involved as proteins active and binding sites.	may potentially interact with the hydrophobic ligands such as lipids and may show specificity for some polar substrates.
		IDG	*Ile* in the IDG motif is involved in recognizing hydrophobic ligands; *Asp* forms stable H-bonds with positively charged amino acids required for proteins stability, and the *Gly* again may provide conformational stability.
		TXXXXXXR	*Thr* is often found in protein centers and capable of forming H-bonds with the polar substrates.
**III**	IIIa	G[ML]G	the *Gly* provides the conformational stability; *Met* and *Leu* play a role in binding and recognition of hydrophobic ligands.	may play an important role in substrate specificity/recognition, specific to aromatic residues, and capable of forming strong H-bonds with the polar substrates.
		GPXTG	*Gly* provides the conformational stability; *Pro* plays an important role in molecular recognition; *Thr* is often	
						found in protein centers and capable of forming H-bonds with the polar substrates.	
		CXXXE	*Cys* may act as a reactive center of an enzyme; *Glu* residues create a stable H-bonds.	
**IIIb**	CXXXQ	*Gln* is involved in protein active and binding sites.
**IV**	-	CDA, FXXXDG, GAAXXXYE, and HXXF	*Ala* is involved in substrate recognition and specificity; *Tyr* makes stacking interactions with the aromatic side chains; *His* is involved in protein metal binding sites; and *Phe* also makes stacking interactions with aromatic side chains.	may show large interactions with the aromatic substrates and these motifs are perhaps involved in substrate recognition and binding.
**V**	-	EDXXH	*His* is most commonly involved in active and binding sites especially in metal binding sites and the *Asp* and *Glu* residues create the stable H-bonds.	may play an important role in reacting with positively charged amino acids.
		GXG	*Gly* provides the conformational stability
**Pog superfamily**	Pog-a	NTL[AT]N	*Asn* is involved as proteins active and binding sites; *Tyr* makes stacking interactions with the aromatic side chains; *Leu* plays a role in binding and recognition of hydrophobic ligands	may play an important role in reacting with hydrophobic ligands and polar substrates
		NXT	Again, *Asn* is involved as proteins active and binding sites; and *Thr* is often found in protein centers and capable of forming H-bonds with the polar substrates.	
		HXXL	*His* is most commonly involved in active and binding sites especially in metal binding sites.	
		RXD	*Arg* is frequently involved in making salt-bridges with the negatively charged amino acids creating stable H-bonds which may be crucial for the structure stability, and *Asp* residues create the stable H-bonds.	
		PXXXFG	*Pro* plays an important role in molecular recognition; and *Phe* is basically involved in stacking interactions with other aromatic side-chains.	
	Pog-b	PRXG	*Pro* plays an important role in molecular recognition; *Arg* is frequently involved in making salt-bridges with the negatively charged amino acids creating stable H-bonds which may be crucial for the structure stability; *Gly* provides the conformational stability.	may be involved in the interaction with aromatic substrates and hydrophobic ligands.
					S[FMV]T	*Ser* is capable of H-bonds with polar substrates; *Met* plays a role in binding and recognition of hydrophobic ligands; and *Thr* is often found in protein centers and capable of forming H-bonds with the polar substrates.
		Pog-c	NTLXN	*Asn* is involved as proteins active and binding sites; and *Thr* is often found in protein centers and capable of forming H-bonds with the polar substrates; *Leu* plays a role in binding and recognition of hydrophobic ligands.	may get involved in making interactions with polar substrates and non-protein ligands.
			PXXGR	*Pro* plays an important role in molecular recognition; *Arg* is frequently involved in making salt-bridges with the negatively charged amino acids creating stable H-bonds which may be crucial for the structure stability; *Gly* provides the conformational stability.	
			S[ML]S	*Ser* is capable of H-bonds with polar substrates; *Met* and *Leu* play a role in binding and recognition of hydrophobic ligands.	
			GWXP	*Trp* may be involved in binding with non-protein ligands.	
		Pog-d	GSLS	*Gly* provides the conformational stability; *Ser* is capable of H-bonds with polar substrates; and *Leu* plays a role in binding and recognition of hydrophobic ligands.	may react with aromatic substrates and hydrophobic ligands.
			RIPY	*Arg* is frequently involved in making salt-bridges with the negatively charged amino acids creating stable H-bonds which may be crucial for the structure stability; *Ile* plays a role in binding and recognition of hydrophobic ligands; and *Tyr* makes stacking interactions with the aromatic side chains.	
		Pog-e	NSLAN	*Asn* is involved as proteins active and binding sites; *Ala* may be involved in substrate recognition or specificity.	may show specificity for some hydrophobic ligands.
			LIXGM	*Ile* and *Leu* is involved in recognizing hydrophobic ligands; *Met* plays a role in binding and recognition of hydrophobic ligands.	
			GLNL	*Gly* provides the conformational stability; *Leu* is involved in recognizing hydrophobic ligands; *Asn* is involved as proteins active and binding sites.	
			HXLI	*His* is involved in protein metal binding sites; *Ile* and *Leu* are involved in recognizing hydrophobic ligands.	
		Pog-f	DXXXFN	*Asp* forms stable H-bonds with positively charged amino acids required for proteins stability; *Phe* makes stacking interactions with the aromatic side chains; *Asn* is involved as proteins active and binding sites.	may show strong structural stability with substrate specificity.
			RXXR	*Arg* is frequently involved in making salt-bridges with the negatively charged amino acids creating stable H-bonds which may be crucial for the structure stability.	

### Database sequences

UPObase is composed of 1948 sequences of UPOs classified into five subfamilies and a superfamily which are subclassified into different sub-subfamilies and sub-superfamilies, respectively (Figure 7). Subfamily I consists of 70 sequences in total including *Aae*UPO categorized into two sub-subfamilies: Ia (54 sequences) and Ib (16 sequences). Subfamily II consists of three sequences. Subfamily III consists of 34 sequences in total categorized into two sub-subfamilies: IIIa (29 sequences) and IIIb (5 sequences). Subfamilies IV and V are not further categorized into sub-subfamilies and consists of 6 and 10 sequences, respectively. The Pog superfamily which consists of the maximum number of sequences (1825 including *Leptoxyphium fumago* and *Marasmius rotula*) in the database is further subclassified into seven sub-superfamilies: Pog-a (90 sequences), Pog-b (47 sequences), Pog-c (26 sequences), Pog-d (17 sequences), Pog-e (8 sequences), Pog-f (128 sequences) and Pog-g (1509 sequences), where Pog-g sequences do not have any signature motif pattern of their own except the *Cys* ligation to the heme which is a characteristic of all HTPs.

## Concluding Remarks and Future Enhancements

We provide a unified platform to analyze all fungal UPOs and UPO-like sequences systematically with easy retrieval and browsing, which can also be successfully used to compare with other enzymes. UPObase also provides a sequence submission portal for new sequences. Besides, it provides a complete classification of UPOs based on their phylogeny and sequence study, and conserved set of sequence motif patterns among these species. UPObase may work as a beneficial tool for the scientists working in the area of fungal UPOs, as it provides annotated data to work on and allows to explore insights to further advance in studying the main physiological role of fungal UPOs. Further developments to UPObase include the better display of homologous searches in the database, search for more UPO and UPO-like sequences and include the protein crystal structures which are currently limited as only two of the fungal UPO protein structures (*Aae*UPO and *Mro*UPO) have been experimentally resolved to date.

## Author Contributions

The original idea of this study was conceived by Y.W., S.H. and D.L. M.F., and S.H. designed the experiments performed by M.F. and collected the data. All authors analyzed the data. The manuscript was drafted by M.F., S.H. and D.L. critically revised by all the co-authors. All authors read and approved the final manuscript.

## Notes

**Database URL:** upobase.bioinformaticsreview.com
